# Retraction: Serological and Entomological Study of Dengue in Dang and Chitwan Districts of Nepal

**DOI:** 10.1371/journal.pone.0270080

**Published:** 2022-06-13

**Authors:** The PLOS ONE Editors

After publication of this article [1], concerns were raised that appropriate ethics approval may not have been obtained for this study according to Nepalese regulatory requirements for research involving human participants. The ethics statement in the article reads, "The ethical committee of Trichandra Multiple Campus, Kathmandu, Nepal approved the consent procedures as well as the study."

The editors discussed this study with the Nepal Health Research Council (NHRC), and they convened a Complaint Handling Sub Committee to examine this matter. The Sub Committee recommended that the article be retracted due to the following:

- The study described in this article [1] required ethical approval under National Ethical Guidelines for Health Research in Nepal. The NHRC confirmed that ethical approval is required for any research involving human participants, including prospective studies and the retrospective use of de-identified samples and medical information collected for the purpose of clinical diagnosis.
- Trichandra Multiple Campus did not have an accredited Institutional Review Committee (IRC) at the time this study was performed, and researchers at this institution conducting research involving human participants were required to obtain ethical approval from the Ethical Review Board of NHRC.
- NHRC did not find a record of this study in the list of proposals approved by the Ethical Review Board.

The corresponding author informed the journal that the study was approved by a committee at Trichandra Multiple College before it was performed. They stated that they no longer have the ethical approval documentation.

As the corresponding author is unable to provide documentation to confirm that ethical approval was obtained for this study, and the NHRC did not find a record of the approval of this study and recommended retraction, the editors consider that this article does not meet *PLOS ONE*’s publication criteria for research involving human subjects. In light of this, the *PLOS ONE* Editors retract this article.

BDP and YS did not agree with the retraction and apologize for the issues with the published article. All other authors either did not comment on this decision, did not respond directly or could not be reached.

## References

[1] Shrestha R, Pant ND, GC G, Thapa S, Neupane B, Shah Y, et al. (2016) Serological and Entomological Study of Dengue in Dang and Chitwan Districts of Nepal. PLoS ONE 11(2): e0147953. 10.1371/journal.pone.0147953 26828951 PMC4735122

